# Swarm dynamics may give rise to Lévy flights

**DOI:** 10.1038/srep30515

**Published:** 2016-07-28

**Authors:** Andrew M. Reynolds, Nicholas T. Ouellette

**Affiliations:** 1 Rothamsted Research, Harpenden, AL5 2JQ, United Kingdom; 2 Department of Civil and Environmental Engineering, Stanford University, Stanford, CA 94305, USA

## Abstract

“Continuous-time correlated random walks” are now gaining traction as models of scale-finite animal movement patterns because they overcome inherent shortcomings with the prevailing paradigm - discrete random walk models. Continuous-time correlated random walk models are founded on the classic Langevin equation that is driven by purely additive noise. The Langevin equation is, however, changed fundamentally by the smallest of multiplicative noises. The inclusion of such noises gives rise to Lévy flights, a popular but controversial model of scale-free movement patterns. Multiplicative noises have not featured prominently in the literature on biological Lévy flights, being seen, perhaps, as no more than a mathematical contrivance. Here we show how Langevin equations driven by multiplicative noises and incumbent Lévy flights arise naturally in the modelling of swarms. Model predictions find some support in three-dimensional, time-resolved measurements of the positions of individual insects in laboratory swarms of the midge *Chironomus riparius.* We hereby provide a new window on Lévy flights as models of movement pattern data, linking patterns to generative processes.

Lévy flights (also known as Lévy walks in the biological literature) are a popular but controversial model of organism movement patterns. They comprise clusters of many short steps with longer steps between them. This pattern is repeated across all scales with the resultant clusters creating fractal patterns that have no characteristic scale^1^. The hallmark of a Lévy flight is a distribution of step lengths with a heavy power-law tail; *p*(*l*) ∼ *l*^−*μ*^ with 1 < *μ* ≤ 3, where *l* is the step-length and *μ* is the power-law (Lévy) exponent (‘∼’ means ‘distributed as’). Widespread interest in Lévy flights as models of movement patterns was ignited by a report that they can be discerned in the flight patterns of wandering albatrosses^2^. Lévy flights as models of movement patterns are, however, controversial, in part because many early studies, including the seminal work of Viswanathan *et al*.^2^, had wrongly ascribed Lévy flights to many species through the use of inappropriate statistical techniques and misinterpretations of the data^3^. Controversy also surrounds the ‘Lévy flight foraging hypothesis’, which posits that because Lévy flights can optimize search efficiencies, natural selection should have led to adaptations for Lévy flight foraging^1^. This hypothesis provided the first conceptual framework for understanding and interpreting Lévy flight movement patterns, but has been fiercely contested^4^. Nonetheless, there is now seemingly compelling evidence that many organisms have movement patterns with Lévy flight characteristics. Lévy flight movement patterns have, for instance, been observed to some extent in the molecular machinery operating within cells^5^, *E. coli* bacteria^6^,^7^, T cells^8^, a diverse range of aquatic marine predators including sharks, turtles and jellyfish^9^,^10^, mussels^11^,^12^, mud snails^13^,^14^, honeybees^15^, human hunter-gatherers^16^, and they have even been observed in trace fossils – the oldest records of animal movements^17^. And it now seems that wandering albatrosses and other seabirds do, after all, roam the high seas in a way that Paul Lévy, after whom Lévy flights are named, would have appreciated^18^,^19^. It has also become apparent that the occurrence of these Lévy flight movement patterns need not be attributed to optimized or advantageous searching^20^.

It is remarkable that flawed data analyses and questionable interpretations have led to a fascinating idea: a general law governing animal movement supplementing that of Brownian motion which underlies the correlated random walk paradigm. Nonetheless, the key to understanding these movement patterns lies with the elucidation of the generative mechanisms rather than *a posteriori* descriptive analysis^21^. Many putative mechanisms have been identified, but most are idiosyncratic and organism specific^20^. The Lévy flight patterns made by honeybees may, for instance, be derived from the Weber-Fechner law in a bee’s odometer^22^. *E. coli* use chemotaxis to locate food but, in the absence of external stimuli, noise in the chemotactic pathways that regulate their ‘run-and-tumble’ locomotion leads to Lévy flight movement patterns^23^. More recently, evidence is accumulating for Lévy flight patterns emerging from chaotic dynamics, albeit with very different origins. The Lévy flight patterns of mud snails have been attributed to neuronal chaos, whilst those in swarming bacteria have been attributed to chaotic flow dynamics^7^,^14^. This is a significant development because chaos is ubiquitous, providing a generative mechanism for Lévy flights that could operate across taxa. The chaotic pathway to Lévy flight patterns in physical systems is well documented, but until recently had not featured in the biological literature^24^. Multiplicative noises provide another general pathway to Lévy flight patterns (sometimes with exponential truncation), one that is well known in the physical sciences but which has yet to feature in the biological literature^25^,^26^.

Here we show that multiplicative noises, and incumbent Lévy flights, arise naturally in the modelling the collective behaviour of swarms. This generative mechanism is a mathematical consequence of swarm dynamics that operates independently of the Lévy flight foraging hypothesis. It also makes no reference to effective social forces, which have often been invoked to explain collective behaviour in animals but have been experimentally elusive to detect^27^,^28^. Model predictions find support in three-dimensional, time-resolved measurements of the positions of individual insects in laboratory swarms of the midge *Chironomus riparius.* Our findings suggest that Lévy flights stemming from multiplicative noise can and do arise inside animal aggregations, including those that, unlike midge swarms, possess strong global ordering.

## Results

### Modelling of midge swarms

Midge swarms do not show the choreographed movement of fish schools or bird flocks, but their members occupy just a small portion of the space available to them^29^. Nonetheless, evidence suggests that individuals are indeed behaving collectively rather than simply interacting independently with an external landmark^29^,^30^. The absence of global ordering makes it a particularly simple system for understanding swarming because coherence which is present in other swarms is not a complicating factor. Here we assume that the positions, *x*, and velocities, *u*, of individual midges can be described by the stochastic differential equations


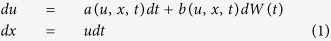


where *dW*(*t*) is an incremental Wiener process with correlation property 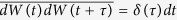. We further assume that the magnitude of the driving noise, *b*(*u*,*x*,*t*), is a constant. Equation (1) is effectively a first-order autoregressive stochastic process in which position and velocity are modelled as a joint Markovian process. At second-order, position, velocity and acceleration would be modelled collectively as a Markovian process. Physically, this hierarchy of models corresponds to the inclusion of a velocity autocorrelation timescale, at first order, and to the addition of an acceleration autocorrelation timescale, at second order, and so on^31^. The first order model, Eqn. 1, is appropriate because midge accelerations are correlated over shorter times than are midge velocities (Supplementary Material). An analogous situation arises when Eqn. 1 has been used to model passive movements in turbulent flows where the neglect of acceleration autocorrelation has been found to be of little practical consequence^31^,^32^,^33^. The deterministic term, *a*(*u, x, t*), is here determined by the requirement that the statistical properties of the simulated positions and velocities be consistent with the observations of Kelley and Ouellette^34^. Kelley and Ouellette^34^ showed that: (1) the spatial distribution of the distance of each individual to the swarm centre is approximately Gaussian in all three dimensions and weakly axisymmetric; (2) and in sufficiently large swarms individual velocity distributions have long, nearly exponential tails.

Mathematically, these consistency conditions require that *a*(*u, x, t*) be a solution of the Fokker-Planck equation


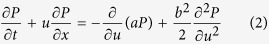


where *P*(*u, x, t*) is the joint distribution of velocity and position^32^. This leads to the classic Langevin equation when velocities are taken to be Gaussian, homogeneous and stationary so that 
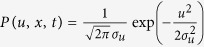
. This equation underlies “continuous time correlated random walk” models of animal movement patterns; models^35^,^36^,^37^,^38^ which overcome inherent shortcomings with discrete correlated random walks that have been the dominate conceptual framework for the modelling of animal movement patterns^39^ Here, in accordance with the observations of Kelley and Ouellette^34^,


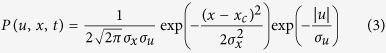


where *x*_*c*_ is the location of the swarm centre, *σ*_*x*_ is the root-mean-square position, and *σ*_*u*_ is the root-mean-square speed. The solution to Eqn. 2 is given by


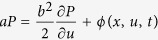


where for statistically stationary swarms having 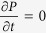,


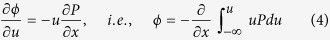


It follows from Eqns 2, 3 and 4 that


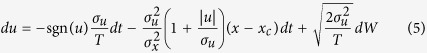


when, without loss of generality, 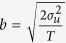 where *T* is a velocity autocorrelation timescale.

Here, it is worth remarking that in small swarms (<10 individuals) velocities are observed to be Gaussian rather than exponentially distributed^34^,^40^, and in this case


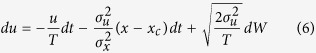


This model is identical to the one posited by Okubo^40^ on the basis that midges act like random moving particles subject to both ‘frictional’ forces that are proportional to velocity and inward ‘cohesive’ forces. In both models, the restoring force increases linearly as the distance from the swarm centre increase, in accordance with observations^34^. Despite their similarities, the two models, Eqns 5 and 6, differ fundamentally when the swarm is liberated from its marker, so that the swarm centre, *x*_*c*_, becomes a dynamical quantity with a stochastic component. The swarm centre is then determined from the instantaneous positions of many simulated midges and so becomes a strongly fluctuating quantity, Here it is tactfully assumed that liberation from the swarm marker does not change the behaviour of individual midges as encapsulated by Eqns 5 and 6. In this case Eqn. 5 has a multiplicative noise term, i.e., a velocity-dependent noise term 
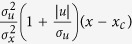
, that may allow for the emergence of heavy-tailed velocity distributions and Lévy flights as it does in other simpler models which are amenable to mathematical analysis^25^,^26^, whilst Eqn. 6 has only additive noise terms. Our model does not appear to be amendable to such analysis. We therefore tested for the emergence of Lévy flights in numerical simulations of midge swarms using Eqn. 5.

The step-length (i.e., flight-segment length) distributions were fitted to truncated power-laws, which are indicative of Lévy flights, exponentially-truncated power-laws, and truncated exponentials (a null model of our movement pattern data):


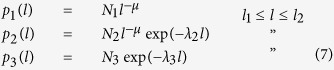


where *N*_1_, *N*_2_ and *N*_3_ are normalisation factors which ensure that the distributions sum correctly to unity when integrated over all time-intervals between the lower and upper cut-offs, *l*_*1*_ and *l*_*2*_; *μ* is the power-law exponent and, *λ*_2_ and *λ*_3_ are exponential decay rates. The lower cut-offs were taken to be start of the tail of the distributions which were estimated by visual inspection of the complement of the cumulative frequency distributions (which we plot). The upper cut-offs were taken to be the lengths of the longest recorded flight segments. Note that cumulative frequency distributions corresponding to truncated power-laws are curvilinear when (as done here) plotted on log-log scales. Fittings were made by maximum likelihood methods^41^,^42^ and the best model distribution was identified using the Akaike information criterion^43^. Data was analysed following the approach of Humphries *et al*.^18^ in which movement patterns are first projected onto the *x*- and *y*-axes to create two one-dimensional movement patterns for each individual. The key idea here is to exploit the fact that the one-dimensional projection of a high-dimensional Lévy flight is itself a Lévy flight. Turns in these projections can then be identified in an unambiguous way as occurring where the direction of travel changes. Without projection turns can only be identified by making reference to arbitrarily defined critical-turning angles. Step lengths were computed on the basis of the one-dimensional sequences and not on the displacements occurring in the two-dimensional sequence between turning points. This projection method was first utilized and discussed in Sims *et al*.^9^ and is now being applied widely^13^,^14^,^17^.

**Figure 1:**
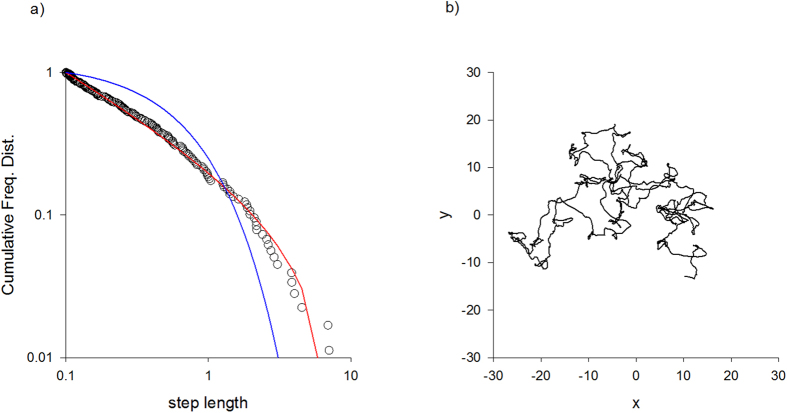
Simulation data produced by the model (Eqn. 5) of midge swarms (10 individuals, σ_x_ = 5.0 a.u., σ_u_ = 1.0 a.u., T = 1 a.u.) (**a**) Complement of the cumulative frequency distribution for the distances travelled between consecutive turns in individual flight patterns (o) together with the best-fit truncated power-law (red-line) and best-fit truncated exponential (blue-line). The maximum likelihood estimate for the power-law (Lévy) exponent is 1.47. The approximate power-law scaling is indicative of a Lévy flight. (**b**) Simulation data illustrating that the swarm centroid moves erratically which in our model leads to multiplicative noise and so to individuals having Lévy flight patterns.

Data from our simulations provide evidence that the movements of individuals within the swarm can be modelled as Lévy flights. Lévy flights are predicted to arise when the midges are flying slowly (or equivalently are flying within large swarms) so that velocity autocorrelations do not persist across distances comparable with the swarm size (i.e., when *σ*_*x*_ ≫ *σ*_*u*_T) (Fig. 1a). These Lévy flights can be attributed to erratic movements of the swarm centroid (Fig. 1b) which in our model leads to multiplicative noise and so to individuals having Lévy flight patterns. Our simulations also indicate that the centres of sufficiently large swarms have Lévy flight movement patterns.

**Figure 2:**
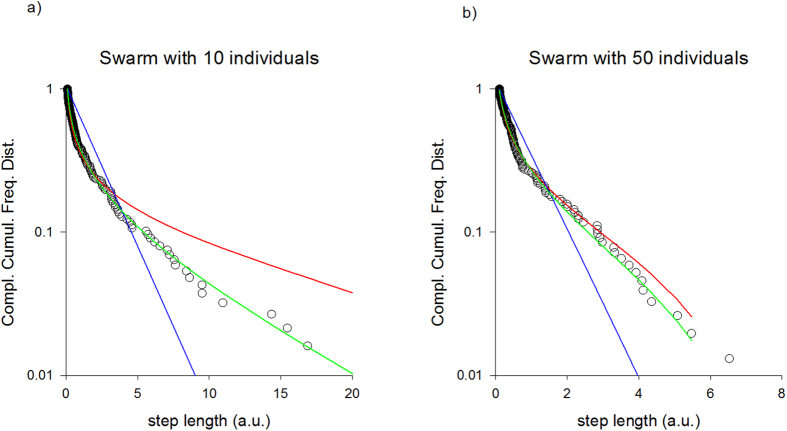
Simulation data produced by the model (Eqn. 5) of midge swarms (σ_x_ = 1.0 a.u., σ_u_ = 5.0 a.u., T = 1 a.u.) with (**a**) 10 and (**b**) 50 individuals. Complement of the cumulative frequency distribution for the distances travelled between consecutive turns in individual flight patterns (o) together with the best-fit truncated power-law (red-line), the best-fit exponentially-truncated power-law (green line) and best-fit truncated exponential (blue-line).

The simulated flight patterns of fast flying midges (or, equivalently, midges within compact swarms) can be modelled as exponentially truncated Lévy flights (Fig. 2).

### Modelling of coherent swarms

**Figure 3:**
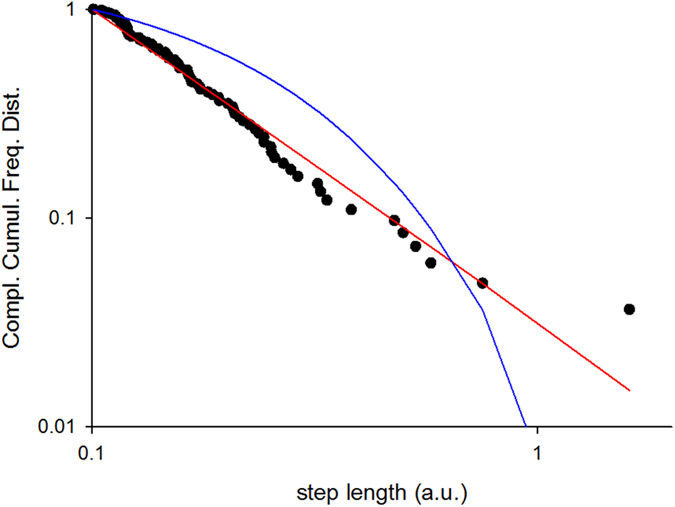
Simulation data produced by the model (Eqn. 8) for 10 interacting individuals with 
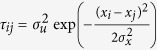
, 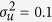 a.u., 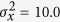 a.u, *b* = *1* a.u. Periodic boundary conditions were used to contain the swarm within a (one-dimensional) box of size 100 a.u. At each time-step 10 randomly chosen pairs of interactions were momentarily turned off. Complement of the cumulative frequency distribution for the distances travelled between consecutive turns in individual flight patterns together with the best-fit truncated power-law (red-line) and best-fit truncated exponential (blue-line). The maximum likelihood estimate for the power-law (Lévy) exponent is 2.49. The approximate power-law scaling is indicative of a Lévy flight.

In contrast with midge swarms, many animal groups (e.g., flocks of birds and schools of fish) possess global order. The modelling framework (stochastic model with Fokker-Planck derived terms ensuring consistency with prescribed velocity statistics) can be extended to take explicit account of interactions between individuals, following the approach of Thomson^44^ who devised a stochastic model for the motion of particle pairs in turbulence. One of the simplest such models is given by


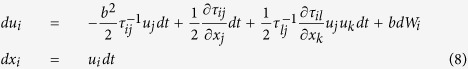


where the subscripts denote different individuals, *τ* is the velocity correlation matrix with components 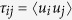, 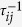 denotes components of *τ*^−1^ and where *dW*(*t*) is an incremental Wiener process with correlation property 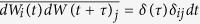. Modelled velocities are Gaussian with mean zero (and close neighbours will have similar velocities by virtue of the correlations). The first term describes how an individual velocity relaxes to a weighted sum of the velocities of its neighbours. The second and third terms ensure that the spatial distribution of individuals is uniform on average. Without these terms, individuals would tend to drift apart because relative velocities tend to decrease as individuals come together and increase as they move apart, leading to a net outward drift, a process akin to turbophoresis. The second and third counter this drift which on average is given by 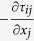. Such velocity-dependent terms are necessarily present if individual velocities are correlated and if average spacing between individuals is constant. The modelling therefore admits the occurrence of Lévy flights when the underlying dynamics are frequently disrupted so that the third term on the right-hand of (Eqn. 8) effectively becomes a multiplicative noise term. This situation would arise, for instance, if some of the correlations were frequently turned off for a short time, so that momentarily *τ*_*ij*_ = 0 for some *i* and *j*’s. This would mimic occasional blocking of the lines of sight between individuals^45^. Here it is tactfully assumed that the introduction of this stochasticity does not change individual behaviour, as encoded in Eqn 8. The results of numerical simulations provide support for this potential pathway to Lévy flying (Fig. 3). Other potential ways of introducing multiplicative noise include restricting the range of correlation, so that the number of interactions (number of conspecifics within the zones of attraction) becomes a fluctuating quantity or making the correlations stochastic to reflect individual uncertainty or inaccuracies in distance estimation.

### Empirical support for the predicted occurrence of Lévy flight patterns

To test the modelling ideas described here in a real animal system, we compared them to empirical data from laboratory mating swarms of the non-biting midge *Chironomus riparius.* Details of the data set have been given elsewhere^27^,^28^,^29^,^34^, and so we describe them only briefly here. We established a breeding colony of *C. riparius* midges in a cubic enclosure measuring 91 cm on a side. Midge larvae developed in nine tanks containing dechlorinated, oxygenated water and a cellulose substrate; upon emergence as adults, midges typically sit on the floor or walls of the enclosure. The midges are exposed to overhead light on a circadian cycle, with 16 hours of light and 8 hours of darkness each day. When the light changes state (corresponding to “dawn” and “dusk”), male midges spontaneously form swarms. To promote nucleation and position the swarms, we provide a 30 × 30 cm^2^ black felt swarm marker in the centre of the enclosure. To measure the motion of the flying midges in the swarms, we image them at a rate of 100 frames per second with three hardware-synchronized Point Grey Flea3 cameras arranged around the enclosure. Using predictive tracking routines originally developed to study intensely turbulent fluid flows^46^, we extract the time-resolved positions, velocities, and accelerations of each midge in the swarms.

**Figure 4:**
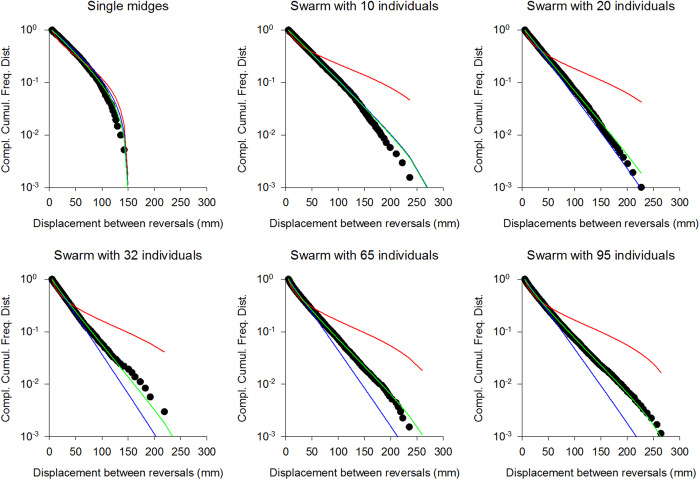
Distributions of horizontal displacements between consecutive reversals for single midges, and swarms with 10, 20, 32, 65 and 95 individuals (•) together with the best fit exponentials (blue lines), best fit exponentially-truncated power-laws (green lines) and the best fit power-laws (red line).

Analysis of the data reveals a gradual transition with increasing swarm size from exponential flight patterns to exponentially-truncated power-law flight patterns (Fig. 4). There is no evidence of Lévy flights *per se* but the flight patterns are clearly very well approximated as exponentially-truncated Lévy flights (Fig. 4), closely mirroring the simulation data for compact swarms (Fig. 2). The absence of Lévy flights *per se* may therefore be the result of the swarms remaining closely bound to their marker. Nonetheless, in accordance with theoretical expectations, flight patterns become increasingly more Lévy-like as the swarm size increases (Figs 2 and 4). This result mirrors the observations of Kelley and Ouellette^34^ who reported, in accordance with the modelling, that the distribution of individual velocities becomes broader as the swarm becomes larger, i.e., as the swarm marker becomes increasingly less important^29^. Further testing of our predictions awaits data for larger swarms.

## Discussion

Many organisms have movement patterns that can be approximated by Lévy flights^5^,^6^,^7^,^8^,^9^,^10^,^11^,^12^,^13^,^14^,^15^,^16^,^17^,^18^,^19^. This accumulation of empirical support is shifting the debate from the question of *can* organisms perform Lévy flights to *how* and *when* they do^47^,^48^. Identification of generative mechanisms is, in fact, crucial because the key to prediction and understanding lies in the elucidation of mechanisms underlying the observed patterns^21^. “Without an understanding of mechanisms, one must evaluate each new stress on each new system *de novo*, without any scientific basis for extrapolation; with such an understanding, one has the foundation for understanding”^21^. This sentiment was recently echoed by Stumpf and Porter^49^ who rightly noted that “a statistically sound power-law is no evidence of universality without a concrete underlying ‘generative mechanism’ to support it”.

Many biologically-plausible putative mechanisms have been identified for the generation of Lévy flight movement patterns^20^; largely absent from the literature, however, has been evidence for general mechanisms that can operate across taxa. The prime candidates are chaos and multiplicative noise. It has been suggested that the Lévy flights of some snails and molluscs can be attributed to neuronal chaos^14^, and that Lévy flights of swarming bacteria can be attributed to their chaotic flow dynamics^7^. Evidence for the multiplicative noise pathway remains elusive, although there is some suggestion that it may operate in some unicellular organisms^50^. Here we provided theoretical evidence that multiplicative noise and incumbent Lévy flights may arise naturally within swarms, as a mathematical consequence of swarm dynamics.

A hallmark of multiplicative noise is a heavy-tailed velocity distribution^25^,^26^. And in this regard it is interesting to note that heavy-tailed velocity distributions have been found to characterize movements of cells in cell aggregates, as well as inert particles in granular matter^51^,^52^,^53^. Cell movements within cell aggregates are also anomalous rather than Brownian with Lévy-like characteristics^51^.

A precursor of our findings can be found in Matsuo *et al*.^54^, who showed that the movement patterns of particles that deform as they propagate can be modelled by the Langevin equation driven by multiplicative noise. They thereby showed how Lévy flights can result from a coupling between deformation and centroid movements. Matsuo *et al*.^54^ noted rightly that their model of ‘active deformable particles’ could find application in the modelling of cell locomotion. Most eukaryotic amoeboid cells, for example, show centroid movement accompanied by large morphological deformations^55^, and there are indications that such cells perform Lévy walks^50^. Nonetheless, the model of Matsuo *et al*.^54^ could also describe collective movements of cohesive groups, a possibility which until now has not been explored. Swarms may in fact provide a more natural setting for the model of Matsuo *et al*.^54^ as they can undergo continuous, significant deformation. Another antecedent of our findings can be found in Reynolds and Geritz^56^ who predicted that fission-fusion dynamics, which are seen for instance in some cell aggregates, can result in multiplicative noise and Lévy flights.

Our findings go beyond those of Matsuo *et al*.^54^ by describing both individual movements within a swarm, and swarm centroid movements, and we go beyond those of Reynolds and Geritz^56^ by breaking free from the reliance of fission-fusion for the emergence of Lévy flights. Our findings thereby open a new window on Lévy flights as models of movement patterns and provide a new perspective on swarming. The occurrence in swarms of Lévy flights is accidental, but may not be without consequence. It could, for example, be of advantage to the male midges when attempting to locate females that have been attracted to the swarm. And the emergence of swarm-centroid movements with Lévy characteristics could benefit the swarm as a whole. In this regard it is interesting to note that the effectiveness of particle swarm optimization codes, a computational approach to solving optimization problems, increases when “individuals” have been programmed to Lévy fly^57^. These advantageous properties stand apart from the contentious Lévy flight foraging hypothesis, in that those assumptions pertain to lone, selfish searchers. Our findings suggest that the programming for within-swarm Lévy flights does not need to be very sophisticated or clever on the individual’s part, as Lévy flight patterns can be derived directly from the swarm dynamics.

## Additional Information

**How to cite this article**: Reynolds, A. M. and Ouellette, N. T. Swarm dynamics may give rise to Lévy flights. *Sci. Rep.* **6**, 30515; doi: 10.1038/srep30515 (2016).

## Supplementary Material

Supplementary Information
