# Supplementary material for: Swarm dynamics may give rise to Lévy flights
Source: Sci Rep. 2016 Jul 28;6:30515. doi: 10.1038/srep30515 (PMC4964348; doi:10.1038/srep30515)
Supplement: Supplementary Information [file srep30515-s1.pdf]

Supplementary Material for “Swarm dynamics may give rise to Lévy flights”

Andrew M. Reynolds[1*] and Nicholas T. Ouellette[2]

[1]Rothamsted Research, Harpenden, AL5 2JQ, United Kingdom. [2]Department of Civil and Environmental Engineering, Stanford University, Stanford, CA 94305, USA.

Second-order modelling

In direct analogy with Eqn. 1, second-order models for the joint evolution of acceleration, A, velocity, u and position, x, take the form

$$
\begin{aligned}
dA &= a(A,u,x,t)dt + bdW(t) \\
du &= Adt \\
dx &= udt
\end{aligned}
\qquad \text{(S1)}
$$

where $dW(t)$ is an incremental Wiener process[33]. The formulation of such models mirrors closely that of first-order models with satisfaction of the well-mixed condition requiring that $a(A,u,x,t)$ be a solution of the Fokker-Planck equation

$$\frac{\partial P}{\partial t} + u\frac{\partial P}{\partial x} + A\frac{\partial P}{\partial u} = -\frac{\partial}{\partial A}(aP) + \frac{b^2}{2}\frac{\partial^2 P}{\partial A^2} \qquad \text{(S2)}$$

where $P(A,u,x,t)$ is the joint distribution of acceleration, velocity and position[32]. Here, in accordance with the observations of Kelley and Ouellette[35], we take

$$P(A,u,x,t) = \frac{1}{4\pi\sigma_x\sigma_u\sigma_A}\exp\left(-\frac{(x-x_c)^2}{2\sigma_x^2}\right)\exp\left(-\frac{|u|}{\sigma_u}\right)\exp\left(-\frac{\left(A-\langle A|u\rangle\right)^2}{2\sigma_{A|u}^2}\right) \qquad \text{(S3)}$$

where x_c is the location of the swarm centre, and where σ_x, σ_u and $\sigma_{A|u}$ are the root-mean-square position, speed, and acceleration. This specification provides a good representation of the position and velocities statistics but under represents the occurrence of large accelerations. An equation for the conditional mean acceleration $\langle A|u\rangle$ is obtained from Eqn. S2 after integrating over all accelerations. For statistically-stationary swarms, with $\frac{\partial P}{\partial t} = 0$, this gives

$$u\frac{\partial P}{\partial x} + \frac{\partial}{\partial u}\left(\langle A|u\rangle P\right) = 0 \qquad \text{(S4)}$$

which has the solution

$$\langle A|u\rangle = -\frac{\sigma_u^2}{\sigma_x^2}\left(1+\frac{|u|}{\sigma_u}\right)(x-x_c) \qquad \text{(S5)}$$

and exactly matches the conditional mean acceleration in the first-order model, Eqn. 1. The conditional acceleration variance is constrained but not determined uniquely by

$$\int \sigma_{A|u}^2 p(u,x,t)du = \sigma_A^2 + \langle A \rangle^2 - \int \langle A|u \rangle^2 p(u,x,t)du$$

which reduces to

$$\int \sigma_{A|u}^2 p(u,x,t)du \approx \sigma_A^2 \qquad \text{(S6)}$$

when, as in turbulent flows[34], σ_A^2 makes the dominate contribution to the right-hand side.

Here, for simplicity, $\sigma_{A|u}$ is taken to be independent of *u*, so that $\sigma_{A|u} \approx \sigma_A$.

It follows from Eqns. S1, S2, S3 and S6 that

$$dA = -\frac{b^2}{\sigma_A^2}\left(A - \langle A|u \rangle\right)dt - \mathrm{sgn}(u)A\frac{\sigma_u}{\sigma_x^2}(x - x_c)dt - \mathrm{sgn}(u)\frac{\sigma_A^2}{\sigma_u}dt + bdW \qquad \text{(S7)}$$

which together with a prescription for *b* defines the second-order model. Here without loss of generality we follow Sawford[33] and let

$$b^2 = 2\sigma_u^2\left(T^{-1} + t_A^{-1}\right)T^{-1}t_A^{-1}$$

where *T* is the timescale introduced at first-order and where t_A is a new, second timescale pertaining to accelerations and defined by $\sigma_A^2 = \sigma_u^2 / Tt_A$.

The second-order model, Eqn. S7, reduces to the first-order model, Eqn. 5, when $t_A / T \to 0$, i.e., when the acceleration timescale, t_A, is much shorter than the velocity timescale. *T*. This can be seen by multiplying both sides of Eqn. S6 by $Tt_A / (T + t_A)$ then taking the limit $t_A / T \to 0$ and by noting that *Adt*=*du*. This gives procedure gives

$$du = -\mathrm{sgn}(u)\frac{\sigma_u}{T}dt - \frac{\sigma_u^2}{\sigma_x^2}\left(1 + \frac{|u|}{\sigma_u}\right)(x - x_c)dt + \sqrt{\frac{2\sigma_u^2}{T}}dW \qquad \text{(S8)}$$

which is identical to Eqn. 5. The first-order model therefore captures many of the properties of the second-order model, especially when $t_A \ll T$. Moreover, the results of numerical simulations (Fig. S1) show that Lévy flights are predicted occur in swarms even when accelerations have significant autocorrelation, e.g., when $t_A = T$.

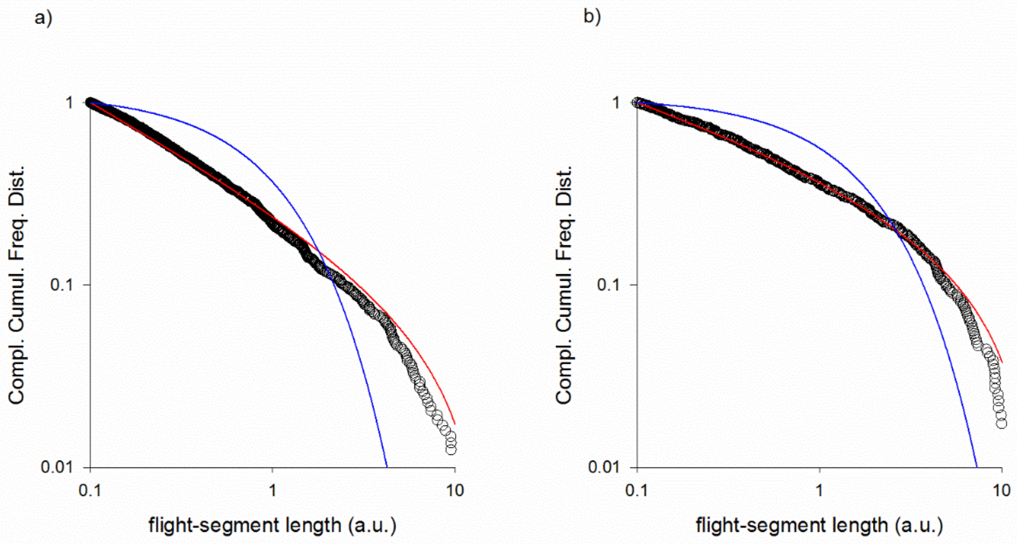

Figure S1 Simulation data produced by the second-order model (Eqn. S7) of midge swarms (100 individuals, σ_x=10.0 a.u. σ_u=1.0 a.u., T=1 a.u.) with **a)** weakly and **b)** strongly auto-correlated accelerations having t_A=0.1 a.u. t_A=1.0. Complement of the cumulative frequency distribution for the distances travelled between consecutive turns in individual flight patterns (o) together with the best-fit truncated power-law (red-line) and best-fit truncated exponential (blue-line). The maximum likelihood estimates of the best-fit truncated power-laws are 1.51 and 1.29. The approximate power-law scaling is indicative of Lévy flight patterns.
